# Successful Pallidal Deep Brain Stimulation Treatment in a Case of Generalized Dystonia due to a Novel *ANO3* Mutation

**DOI:** 10.1155/2019/3154653

**Published:** 2019-11-29

**Authors:** Lizl Lasky, Lindsay Bliss, Christos Sidiropoulos

**Affiliations:** Department of Neurology and Ophthalmology, Michigan State University, 804 Service Rd. B-411, East Lansing, MI 48824, USA

## Abstract

**Background:**

Dystonia is a ubiquitous syndrome, with a growing number of genes being continually identified. Mutations in the anoctamin-3 gene have been described to cause dystonia but the management and long-term outcomes are still largely unknown.

**Methods:**

We present here a long term, longitudinal follow up of a patient with generalized dystonia, who was treated with bilateral pallidal deep brain stimulation and was found to harbor a mutation in the anoctamin-3 gene.

**Results:**

Ongoing adjustment of stimulation settings and medications led to good and sustained dystonia control; however the patient did suffer short term relapses, manifested as dystonic crisis, which necessitated inpatient admission.

**Conclusion:**

This only the second patient to be reported with pallidal stimulation and an anoctamin-3 gene mutation. Long term outcomes seem to be favorable but larger case series are needed to confirm our findings.

Dystonia is a ubiquitous group of movement disorders characterized by excessive muscle contraction which leads to abnormal movements and/or postures. Research focus has been on finding underlying genetic causes, such as in DYT-TOR 1A and DYT-THAP 1. More recently, mutations in the anoctamin-3 gene (*ANO3*, OMIM 610110, DYT 24) have also been implicated as the cause of autosomal dominant dystonia. The phenotypic spectrum of this condition is broad, ranging from cranio-cervical dystonia [1] with tremor, upper limb dystonia [2], lower limb dystonia [3], blepharospasm, myoclonus dystonia, and dystonia-parkinsonism [4] typically with age at onset between the 3^rd^ and 5^th^ decade of life [5], although children with *ANO3* mutations have been described. The presence of tremor may help differentiate *ANO3* from dystonia due to mutations in the *GNAL* gene. In most patients, *ANO3* related dystonia progresses slowly over many years. Incomplete penetrance may be another characteristic. To our knowledge, there is a paucity of reports of generalized dystonia as the presenting symptom, mostly occurring in the pediatric population [3, 6].

Deep brain stimulation (DBS) of the globus pallidus pars interna (GPi) has been proven effective in treating dystonia poorly responsive to medications, although data is focused on DYT-TOR1A, and DYT-THAP1. We present a patient with generalized dystonia due to a novel mutation in the *ANO3* gene and the long-term response to DBS. This is, to our knowledge, only the second genetically confirmed case of *ANO3* dystonia to be treated with DBS, besides the one briefly mentioned in the paper by Olschewski et al. [5].

Our patient is a 46-year-old female who developed generalized dystonia at age 19, affecting primarily the lower extremities, and then spreading to the cranio-cervical region, severely affecting her ambulation. Dystonic storms would occur every few weeks. In 2012 she underwent deep brain stimulation of the bilateral globus pallidus interna (GPi) after having failed traditional medical management for dystonia, which consisted of adequate doses of oral baclofen and botulinum toxin injections in the lower extremities, with a Medtronic Activa PC (Medtronic Inc., MN, USA) implantable pulse generator (IPG), and a 3387-lead model. Since then she has had significant and persistent improvement in her dystonia with practically no residual symptoms during most of the days. Her current DBS settings are left GPi: C+ 0-1− 3 V/180 *µ*s/60 Hz and right GPi: C+ 8-9− 3.5 V/180 *µ*s/60 Hz. Eventually, due to high energy consumption IPG was converted to a rechargeable Activa RC by the same company. The patient has over time developed mild and tolerable bilateral upper extremity bradykinesia, felt to be due to DBS, as described by others in the literature [7].

Since DBS placement, and over a period of 5 years, she has had 5 episodes of dystonic storms during which she required hospitalization with frequent and high dosing of IV benzodiazepines, exceeding 40 mg of diazepam daily as well as 60 mg of baclofen, and 4 mg of benztropine. Following these hospitalizations, she has been discharged on additional benzodiazepines which were weaned down as an outpatient. Her current maintenance regimen is baclofen 20 mg three times daily, benztropine 1 mg thrice daily, diazepam 5 mg as needed three times daily for breakthrough symptoms, and duloxetine 60 mg daily. After her last hospital admission in 2018 a targeted 21 gene panel for various forms of dystonia by Invitae Inc (Invitae, San Francisco, CA) revealed a novel mutation in the *ANO3* gene, c743A>C (pGln248Pro). This variant is not present in population databases, like ExAC or 1000 Genome Project. It is predicted to be disease causing by Mutationtaster (http://mutationtaster.org). It is notable to mention that from April of 2018 until the writing of this manuscript (November 2019) the patient has not had any episodes of dystonic storms, whatsoever. At present, she has no limitations at all in her ambulation and hand dexterity and practically, minimal to none, dystonic symptoms.

The exact function of anoctamin-3 is unknown, although reports suggest it is a probable Ca+2-activated chloride channel. It does not produce a robust current, however, indicating that it is likely more involved in intracellular processes. *ANO3* is also involved in regulating Ca homeostasis and modulating neuronal excitability. The putamen is the highest *ANO3* expressing region, followed by the frontal cortex. As a result, abnormal neuronal excitability resulting from abnormal *ANO3* may present clinically as dystonic movements.

Most of the published research regarding DBS as a therapeutic strategy for dystonia has focused on primary generalized dystonia like DYT-TOR1 or DYT-THAP1 and cervical dystonia. GPi has been the preferred target thus far with the therapeutic effect thought to involve modulation of thalamocortical outputs and normalization of motor cortex hyperexcitability. Not all patients respond equally. Factors that predict a better outcome in isolated dystonia patients include younger age at surgery, shorter duration of disease, lower baseline motor severity, and mutations in the *TOR1A* gene. *TOR1A* mutations respond more consistently than *THAP1* mutations and genetic testing may have a value in predicting motor outcomes in dystonia. Our patient is the second one harboring an *ANO3* mutation with good and long term (7 years) results from pallidal DBS. Inspite of the clinically meaningful response to DBS, the addition of oral medications as well as ongoing adjustment of her neurostimulation parameters had to be undertaken to control her symptoms, and even so she had significant breakthrough events that required hospitalization.

However, the patient's quality of life improved great after surgery and she was able to have essentially normal motor function in between episodes.

This builds on the evidence so far that monogenic dystonia, poorly controlled on medications, tends to respond favorably, although not completely, to DBS.
